# DTWscore: differential expression and cell clustering analysis for time-series single-cell RNA-seq data

**DOI:** 10.1186/s12859-017-1647-3

**Published:** 2017-05-23

**Authors:** Zhuo Wang, Shuilin Jin, Guiyou Liu, Xiurui Zhang, Nan Wang, Deliang Wu, Yang Hu, Chiping Zhang, Qinghua Jiang, Li Xu, Yadong Wang

**Affiliations:** 1 Department of Mathematics, Harbin Institute of Technology, Harbin, Heilongjiang, 150001 West Dazhi Street China; 2 School of Computer Science and Technology, Harbin Institute of Technology, Harbin, Heilongjiang, 150001 West Dazhi Street China; 3 College of Computer Science and Technology, Harbin Engineering University, Harbin, Nantong Street, Heilongjiang, 150001 China; 4 School of Computer Science and Technology, Harbin Institute of Technology, Harbin, Nantong Street, Heilongjiang, 150001 China

**Keywords:** Single-cell RNA-seq, Time-series data, Dynamic time warping

## Abstract

**Background:**

The development of single-cell RNA sequencing has enabled profound discoveries in biology, ranging from the dissection of the composition of complex tissues to the identification of novel cell types and dynamics in some specialized cellular environments. However, the large-scale generation of single-cell RNA-seq (scRNA-seq) data collected at multiple time points remains a challenge to effective measurement gene expression patterns in transcriptome analysis.

**Results:**

We present an algorithm based on the Dynamic Time Warping score (DTWscore) combined with time-series data, that enables the detection of gene expression changes across scRNA-seq samples and recovery of potential cell types from complex mixtures of multiple cell types.

**Conclusions:**

The DTWscore successfully classify cells of different types with the most highly variable genes from time-series scRNA-seq data. The study was confined to methods that are implemented and available within the R framework. Sample datasets and R packages are available at https://github.com/xiaoxiaoxier/DTWscore.

**Electronic supplementary material:**

The online version of this article (doi:10.1186/s12859-017-1647-3) contains supplementary material, which is available to authorized users.

## Background

Methodological advances provide transcriptomic information on dozens of individual cells in a single-cell sequencing project [1–3] to study the complex cellular states and to model dynamic biological processes [4]. From traditional bulk samples RNA sequencing (RNA-seq) to single-cell RNA sequencing (scRNA-seq), cell-to-cell variabilities expose latent biological characteristics such as cell cyclic processes [5] and transcriptional heterogeneity [6], that disappears with bulk gene expression across thousands of cells. Additionally, biological processes are often dynamic, while bulk RNA-seq data may blur heterogeneity [6] and un-synchronization [7] of the transcriptional process. These features can be well represented owing to the advent of scRNA-seq of sequential gene expression changes, which provides a set of time slices from individual cells sampling from different moments in the process [8]. Developments in techniques for measuring gene expression [9] make time-series expression studies more feasible with the relative database growing exponentially [10]. Nonetheless, profiling the low amounts of mRNA within individual cells leads to several experimental and computational challenges such as so-called ‘dropout’ events [11], which involve the false quantification of a gene as ‘unexpressed’ because of the corresponding transcript being ‘missed’ during the reverse-transcription step [12]. This occurrence leads to a lack of detection during sequencing, which is observed in scRNA-seq measurements with lower expression magnitudes. Moreover, with different types of temporal response patterns observed in biological processes, identifying the set of genes that participates in specific response also poses a challenge for advanced computational methods [13].

Among others, one key objective is to define the sets of genes that best discriminate transcriptional differences by inferring the heterogeneity of cells’ unsynchronized evolution [14]. This strategy is important for discovering multiple cell fates stemming from a single progenitor cell type [15]. In essence, with each cell collected at a distinct time point, scRNA-seq experiments would constitute a time series through a biological process by ordering single-cell expression profiles in multiple time points [15]. Hence, time-course measurements with time-series gene expression data benefit researchers by capturing focused genes with transient expression changes [16]. We show an unsupervised approach to infer heterogeneity using time-series data derived from unsynchronized differentiation cells, rather than relying on known marker genes or experiments starting from synchronized cells within a quantitative measure of progress. Then we cluster complex mixture of single cells based on these highly divergent genes to define potential cell types. In the context of bulk RNA-seq, many popular tools for differential expression analysis are used [17–19]. However, these methods simply compare gene expression levels between groups, a process that is not suitable to manage time-series scRNA-seq data. By contrast, the key approach for scRNA-seq data analysis is based on dimensional reduction. SLICER [8] makes use of a nonlinear dimensionality reduction algorithm to capture highly nonlinear relationships between gene expression levels. Monocle [15] infers a low-dimensional manifold embedded in a high-dimensional space that obtains the observed geometric relationships among the cells. Other than dimensionality reduction, Wanderlust [20] can capture nonlinear behavior through finding the shortest paths by *k*−nearest neighbor graphs without dimensional reduction. Critically, dimensional reduction does not make full use of the rich information provided by scRNA-seq time-series data. However, the existing methods may overlook un-synchronization over the entire time series. It is a challenging problem to provide the approaches to identify the set of genes from distinct cells that are differentially expressed over time. Moreover, estimating at which time periods the transcriptional heterogeneity with different cell types is present can provide additional insight into temporal gene functions.

In this article, we present an algorithm based on the Dynamic Time Warping score (DTWscore) [21] that is used in scRNA-seq time-series data to infer the potential cell types between time period the first time. DTWscore provides three significant advantages for inferring the potential cell types: (1) It is capable of managing unevenly and sparsely sampled time-series gene expression data without need for prior assumptions about the evenness or density of the time-series data; (2) the method uses dynamic time warping (DTW) algorithms to consider the similarity of pairs of vectors taken from each time series between the gene expression levels and progression through a process. The DTWscore shows the classification of potential cell types and corrects for synchronization loss; (3) The method is capable of maintaining the sensitivity and specificity with scRNA-seq gene expression data that has been tested in various experimental designs.

## Results

### Overview of the DTWscore method to detect highly divergent genes and classify potential cell types from time-series scRNA-seq data

For single-cell RNA-seq data, the gene expression level of some fixed time points become more easily obtainable than traditional bulk RNA-seq data [8]. A commonly used method for assessing the variability is the ratio of the fold-change [22], calculated as the ratio between the mean expression values of samples, which illustrates its limitation in dealing with time-series data. To overcome the deficiency, we implemented DTW algorithms on synthetic and real time-series scRNA-seq data. DTW was originally developed for speech recognition in the 1970s [23]. Similar to the algorithms used for sequence comparisons, the DTW algorithm is particularly suitable for identifying highly variable genes between scRNA-seq time-series data especially unsynchronized time-course data. In several time-series experiments, cells may not be synchronized over the entire time series, while these cells may be involved in the same cyclic progress. For each gene, its expression values from different time points represent the biological process. Whether or not one gene is involved in the different biological processes between different cell samples or diverse tissues is essential for characterizing the heterogeneous genes. Each gene is given an average DTWscore based on its time-series expression levels from all pairs of cells, and a threshold based on the distribution of all the DTWscores is set to choose the specific genes that present the significantly variable progression. Cells could be clustered based on the highly divergent genes to define potential cell types. To demonstrate the performance of the DTWscore, we applied it to several simulated examples and public datasets with new biological insights.

**Fig. 1:**
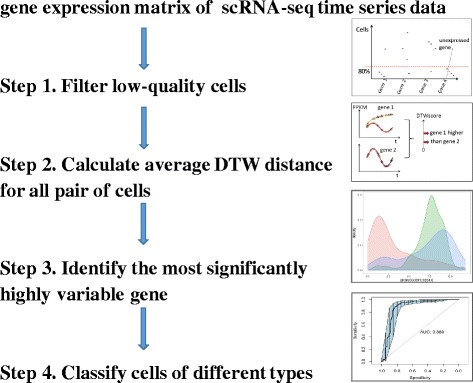
Overview of the DTWscore pipeline. Details are described in the Methods

Briefly, the DTWscore focuses on detecting the cell-to-cell heterogeneity among time-period scRNA-seq data and highlights the highly divergent genes that are used to define potential cell types. The input of the DTWscore is a matrix of time-series gene expression data. The rows of the matrix stand for individual genes, and the columns represent the gene expression profiles of different cells from discrete time points. The method is performed on both simulated and real datasets. In particular, if a gene expression level between different time periods is quantified through the same process function, we consider genes of this type to show non-heterogeneity across cells, while the remaining genes are deemed as highly variable genes between time series data for further analysis. A graphical representation of our method pipeline is displayed in Fig. 1. First, we performed the traditional filter step to filter low-quality cells. To identify poor-quality libraries from further analysis, we hold the identifiers for genes expressed in at least 80 percent of total cells in the data set. Second, we calculated the mean DTW distance of all pair of cells as the index for detecting a specific set of genes for heterogeneity analysis. Based on the DTW distance index, we normalized the DTW index values to reduce the bias toward extreme values. After normalization, the gene with the highest DTWscores are selected for further analysis and are referred to as the most significantly highly variable gene. The Flexible threshold for choosing the sets of genes can be adjusted by the normal distribution of the all the average DTWscores for each gene. The output of our result could be used for classifying cells of different types. Furthermore, some heterogeneous genes could serve as potential biomarkers that track some disease processes. The details of the DTWscore pipeline are described in the Methods section.

### The DTWscore identifies differentially expressed genes from time-series scRNA-seq data

#### Synthetic time-series scRNAseq data

**Fig. 2:**
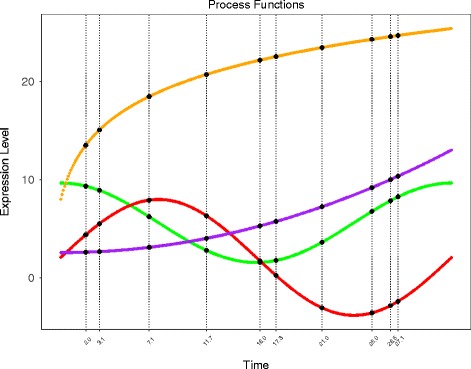
Simulated trajectories of gene expression levels over time. The x-axis represents time and the y-axis represents FPKM values of gene expression. The genes are represented by four types of continuous curves that highlight the dynamics of expression changes

**Fig. 3:**
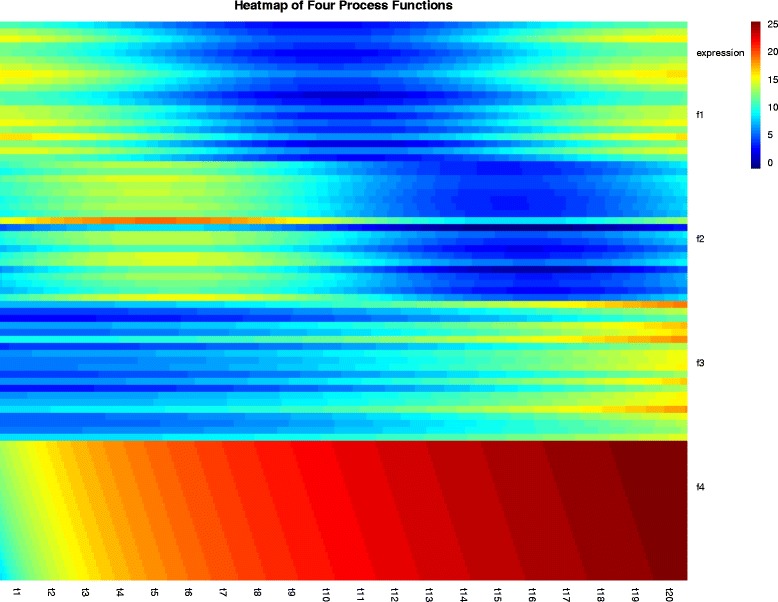
Four patterns of gene expression for each functions. A more precise overview of different gene expression process in time order. Heatmap shows gene expression levels from samples that were taken at even time intervals. Experiments shows four pattern of gene expression for each functions

We borrowed functions [8] with a ‘process time’ parameter *t* to simulate gene expression patterns with four different ‘biological processes’(see Methods for details). If the gene expression patterns are tracked during the unfolding of a biological process, the process can be conceived as some specific functions over time. Four typical trajectories of gene expression are simulated graphically (Fig. 2). Heat maps are a popular way to display gene expression levels. As shown in Fig. 3, heat map is plotted with equal width for each time points to make an external direct-viewing impression on the time-series gene expression data. The input of the heatmap is a matrix whose rows represent the four types of process functions and columns represents the discrete time points.

**Fig. 4:**
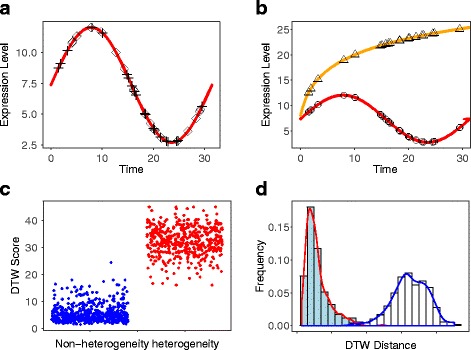
DTWscore identifies heterogeneous and non-heterogeneous genes from the synthetic data. **a** Temporal patterns of gene expression from a single biological function. *Diamonds* and *crosses* shows the time points at which samples were collected from the two time periods. Samples were taken at uneven time intervals. **b** Temporal patterns of gene expression from two biological functions. *Triangles* and *circles* show the time point at which samples were collected from the two time periods. **c** Jitter plot of DTWscore between non-heterogeneous genes versus heterogeneous genes, displaying clear clusters. **d** Bins in the horizontal axis summarize changes in the overall expression group of bars corresponding to genes from simulated datasets. Colored bars within each group summarize changes in DTW distance between groups. The figures show that the DTWscore is effective for identifying gene expression patterns

**Fig. 5:**
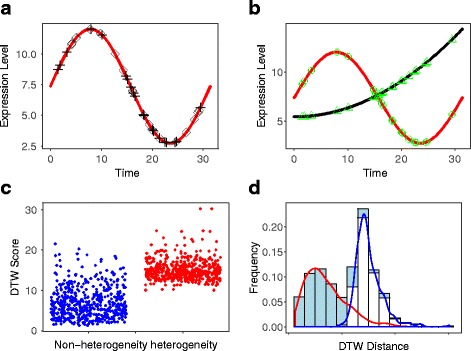
DTWscore identifies heterogeneous and non-heterogeneous genes from synthetic data. **a** Temporal pattern of gene expression from a single biological function. *Diamonds* and *crosses* shows the time points at which samples were collected from the two time periods. Samples were taken at uneven time intervals. **b** Temporal patterns of gene expression from two biological functions. *Triangles* and *circles* show the time points at which samples were collected from the two time periods. **c** Jitter plot of DTWscore between non-heterogeneous genes versus heterogeneous genes, displaying clear clusters. **d** Bins in the horizontal axis summarize changes in the overall expression group of bars corresponding to genes from simulated datasets. Colored bars within each group summarize changes in DTW distance between groups. The figures show that the DTWscore is effective for identifying gene expression patterns

**Fig. 6:**
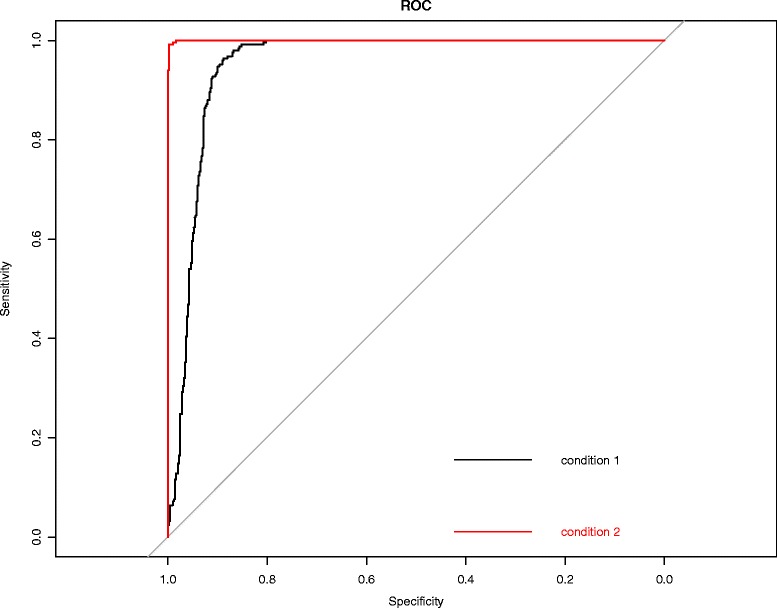
ROC curves from different conditions. The DTWscore method was applied to two different scRNA-seq time series data sets. The algorithm’s performance was assessed by their sensitivity, illustrated in the ROC curves, which demonstrate good performance in all cases. The *black curve* represents condition 1 simulated by the biological functions *f*_2_(*t*) and *f*_3_(*t*). The *red curve* represents condition 2 simulated by the biological functions *f*_2_(*t*) and *f*_4_(*t*)

In the simulation, two groups of scRNA seq data with time are generated as follows. Group one (non-heterogenerous genes): the gene expression matrix at multiple time points is generated by the same function shown in Fig. 2, indicating that this gene undergoes the same biological process. Group two (heterogeneous genes): the gene expression matrix at multiple time points is generated by different functions shown in Fig. 2, indicating that this gene undergoes different biological processes. Additionally, the number of time points could be the same or different, which is a good feature of DTW algorithms. More details regarding the setup can be found in the‘Methods’section. To address the issue with identifying differentially expressed gene patterns in scRNA-seq data and classifying different cell types, we perform the DTWscore pipeline on synthetic datasets under six conditions (Figs. 4 and 5, Additional file 1: Figure S1, Additional file 2: Figure S2, Additional file 3: Figure S3 and Additional file 4: Figure S4). The simulated dataset consists of the two groups of 1000 gene expression levels with two time periods. In group one, 500 genes undergo the same biological process between two time periods and their expression values are simulated by a single family of functions. In group two, 500 gene’ expression values are generated from different families of functions. We compute the average DTWscore to identify genes that were from the same biological processes or heterogeneous processes, as shown in Figs. 4 and 5. After normalization for the origin DTW index, high DTWscore genes are enriched in the group of genes that are simulated by different families of process functions. Figure 4 c and d show that the DTWscores are clustered from different gene sets. The DTWscore algorithm successfully identified time-series genes of non-heterogeneity versus heterogeneity. We performed DTWscore analysis on various synthetic datasets and repeated the analysis times, and the results suggest that the DTWscore performs well in the analysis (Figs. 4 and 5). Next, we evaluated the discriminative power of the DTWscore in terms of receiver operating characteristic (ROC) curves, using two simulating datasets labeled conditions 1 and 2. In particular, for the comparison, genes are divided into a true-positive group and a true-negative group according to the simulating strategy. Thereafter, ROC curves were constructed by calculating the true and false positive rates for all possible thresholds (Fig. 6). The black curve represents condition 1 simulated by the biological functions *f*_2_(*t*) and *f*_3_(*t*), while the red curve represents condition 2 simulated by the biological functions *f*_2_(*t*) and *f*_4_(*t*).

### Highly divergent genes define the potential cell types from time-series scRNA-seq data

#### Human skeletal muscle myoblasts (HSMM) data

**Fig. 7:**
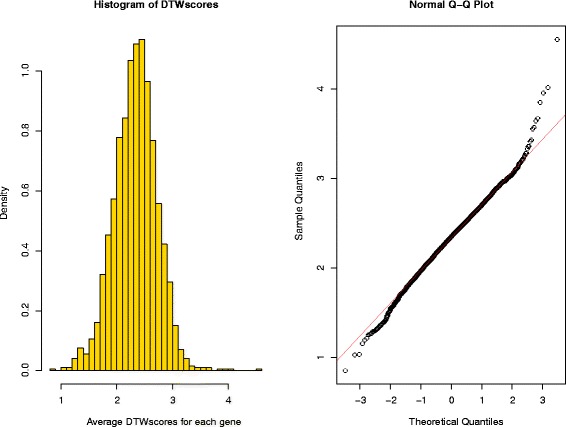
Histogram and Q-Q plot of DTWscore based on HSMM datasets. The histogram plot of DTWscores shows that the distribution of values is normally distributed. The linearity of the points in the Q-Q plot is the best proof. Meanwhile, outlier of the distribution appears on the right corner. Thus, genes with DTWscore more than 4 standard deviations above the mean are considered heterogeneous genes

**Fig. 8:**
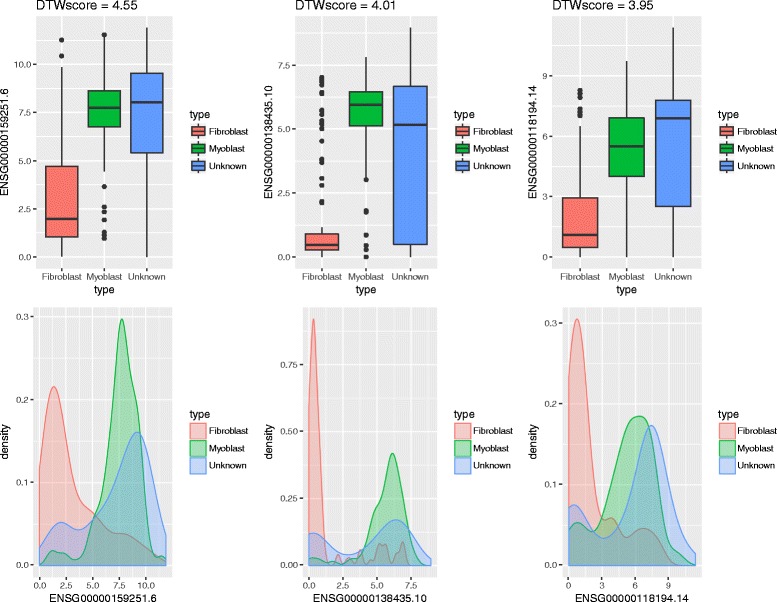
Genes with the top three DTWscores. The boxplot and density represent temporal gene expression values of three highly variable genes from all pairs of cells. It is obvious that these genes should be declared as differentially expressed. The genes with highest DTWscores undergo different expression pattern and play an important role in the following clustering analysis

**Fig. 9:**
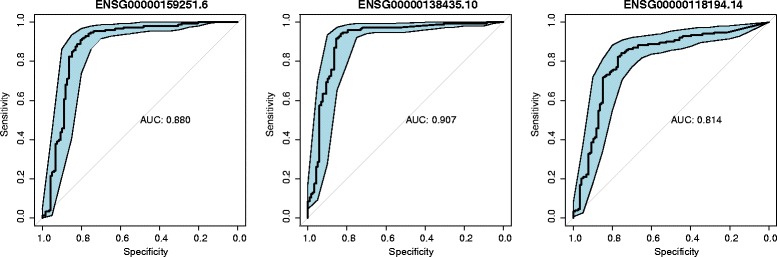
Receiver operating characteristic (ROC) curves for prediction. Receiver operating characteristic (ROC) curves for classification made by the most highly variable gene. Also shown are ROC curves for classification made by the other two genes chosen by the DTWsocre. This function computes the confidence interval (CI) of the sensitivity at the given specificity points. By default, the 95% CI are computed with 2000 stratified bootstrap replicates

In this section, DTWscore is applied to the recently published data from human skeletal muscle myoblasts [15] which were captured between single cells at four time points. The data were generated from RNA from each cell, which was isolated and used to construct a single mRNA-seq library per cell with a sequencing depth of ∼4 million reads per library. The fragment per kb per million mapped fragments (FPKM) expression profiles are provided on the Gene Expression Omnibus (GEO) website. Our goal is to classify cells from complex mixtures of multiple cell types and investigate the cell-to-cell heterogeneity between two time periods by identifying the highly variable gene expression patterns. Differentiation across a set of cells proceeds at potentially different rates, relying strictly on the time points. With the collected data at different time points, we would like to determine a set of genes exhibiting variabilities across cells with the same or different time periods. Our method was different from the traditional cell cluster detection methods with no need for biological prior knowledge. We sought to identify the most highly divergent genes that could be used to define potential differentiation states. All pairs of cells were chosen from this group of cells based on two time period and we calculate each genes’ average DTWscores for all pairs of cells. As shown in Fig. 7, the histogram displayed the density of the DTWscores which obeys a Gaussian distribution. The Q-Q plot in Fig. 7 compares the data generated by DTWscores on the vertical axis to a standard normal population on the horizontal axis. The linearity of the points suggests that the data are normally distributed. We could make full use of the mean and the standard deviation of the Gaussian distribution to determine the highly variable genes. Owing to the distribution of DTWscores, we take the 4 standard deviations above the mean as the threshold for identifying heterogeneous genes (Fig. 8). Three genes with the top three DTWscores 4.55, 4.01 and 3.95 show a significant difference between cell types myoblasts and fibroblasts. We plot the expression levels of these genes by boxplots and density plots (Fig. 8), to better highlight the differences between cell states. Hence, without any biological knowledge, we have selected the possible marker genes that tend to be highly informative about cell states and types. Moreover, we analyzed three genes with the highest DTWscores for model-based clustering. With the two covariance structures, finite Gaussian mixture model provides functions for parameter estimation via the expectation maximization (EM) algorithm (Fig. 9). We simply call *Mclust* function from R package ***mclust*** [24, 25] to perform cluster analysis of the three genes respectively. Receiver operating characteristic (ROC) curves for predictions (Fig. 9) shows the good performances of our classification. We computes the confidence interval (CI) of the sensitivity at the given specificity points. Moreover, two genes or three genes might also be driving the clustering (Additional file 5: Figure S5 and Additional file 6: Figure S6).

## Comparison with other methods

In order to assess the performance of DTWscore in relation to other approaches, we run Monocle and SLICER on the HSMM data and compared the classification results from all the three approaches.

**Fig. 10:**
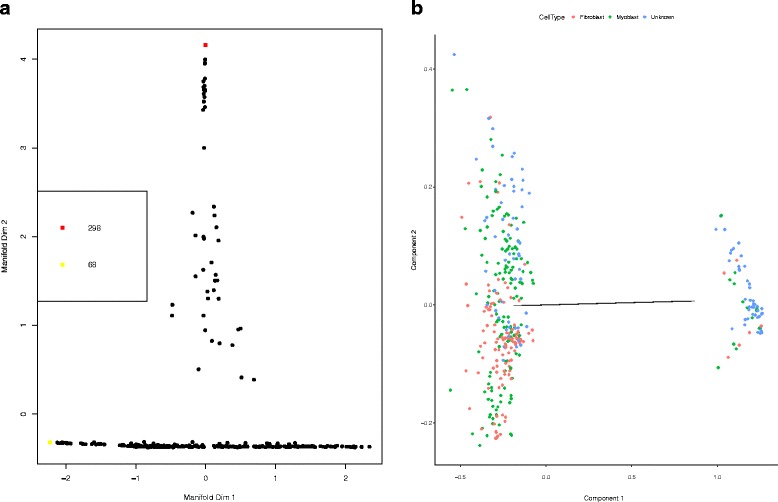
SLICER and Monocle results from HSMM data. **a** Cellular clustering inferred by SLICER. Cells colored according to the branches that SLICER assigned using geodesic entropy. **b** Cellular clustering inferred by Monocle. The cells tagged as myoblasts are marked in *green*, while the fibroblasts are tagged in *red*

**Fig. 11:**
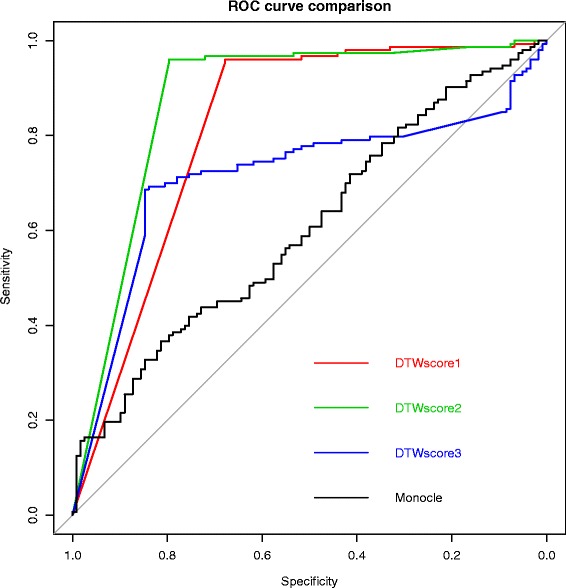
Receiver operating characteristic (ROC) curves for comparison with Monocle. Genes with the top three DTWscores are chosen for cells cluster respectively. ROC was performed for comparing with Monocle method which shows the better performance of DTWscore method

Monocle uses independent component analysis (ICA) to reduce the dimensionality of the expression data before clustering the cells. Monocle also provides algorithms on unsupervised cell clustering and semi-supervised cell clustering with known marker genes. Figure 10 b shows that the cells fall into two different clusters. The cells tagged as myoblasts are marked in green, while the fibroblasts are tagged in red. Unfortunately, the cells don’t cluster by type. This is not surprising because myoblasts and contaminating interstitial fibroblasts express many of the same genes in these culture conditions. While DTWscore method makes full use of the information between all pairs of cells by calculating time series DTWscores. This process help DTWscore infer the most stable marker genes for defining the potential cell types. Figure 11 shows the roc curves for the comparison between DTWscore and Monocle methods which present the better performance of DTWscore method.

Because SLICER can infer highly nonlinear trajectories and determine the location and number of branches and loops, the cells fall on more different branches. Figure 10 a is the default low-dimensional *k*-nearest neighbor graph shows the clustering using SLICER branching analysis. It appears that SLICER benching analysis suggest that cells should fall on many different branches which maybe more than the real number of cell types. Obviousily SLICER is capable of detecting types of features but sometimes it will overfit. However, DTWscore is a model-based method to infer the potential cell types which is more flexible for diverse datasets.

## Discussion

We stress that our method is different from the approach that detects cell clusters and expression differences, such as those described previously [8, 15, 20], which seek to infers cellular trajectories from scRNA-seq data. In addition to identifying differentially expressed genes from the time series data, our framework allows us to identify potential cell types that undergo differetiation at each time point. Such genes are of great interest. First, they represent biological heterogeneity within heterogeneous cells, implying differential regulation of response across cells. Second, these genes could be used for marker genes to distinguish from mixture of cell types. Finally, we hypothesize that heterogeneous genes can serve as biomarkers that track the progressive disease process. If confirmed, our study will discover and monitor disease processes prior to the onset of clinical symptoms. We also do not require dimensionality reduction with many important genes going unobserved. The real strength in our framework lies in the capacity to characterize the potential cell types by inferring differentially expressed genes, which provides the opportunity to study the extent of gene-specific expression heterogeneity within a biological condition.

The approach is limited in that only classification of cell types are feasible. A generalized DTW algorithm used for the analysis will make analyses of more than three to four cells over time possible; work in that direction is underway. Finally, we note that, while the differentially expressed genes identified by the DTWscore may prove useful in downstream analysis and cellular branches and trajectories inference, extensions in this direction are also underway.

## Conclusions

To date, a large amount of available high-throughput data has been measured at a single time point [26]. Time-series expression experiments provide a wealth of information regarding the complete set of gene expression patterns [27]. Thus, a large body of literature has integrated these temporal data sets using computational methods [28–30]. Meanwhile, many quantitative tools have sought to [31–33] study changes in gene expression and the potential cell states at the single-cell level. For a better understanding of the single-cell expression level combined with time-series data, we focused on the detection of genes whose biological heterogeneity varies between cells and inferring the potential cell types from complex mixtures of multiple types. This analysis is quantified with our proposed DTWscore, which is used as the basis to select highly variable genes. According to the experimental results, the DTWscore is effective with cell type clustering based on single-cell expression time-series data.

Our analysis of scRNA-seq time-series gene expression datasets increased the ability to study various cellular mechanism over time. First, in HSMM cells, we identified highly significantly differentially expressed genes with time-series data, indicating that the genes are marked for use in the following clustering. The expression of these genes possibly arose from the un-synchronized time-series scRNA-seq experiments. Second, given the various biological processes, the DTWscore for each gene was calculated using our pipeline. By combining the method to set thresholds, quantitative analysis has enabled the direct separation of heterogeneous and non-heterogeneous genes. The DTWscore can manage uneven and sparsely sampled time series gene expression data without need for prior assumptions about the evenness or density of the time-series data. Moreover, all pairs of cells are calculated by DTWscore, a procedure that could result in the stability of finding important highly variable genes. Finally, the DTWscore could successfully identify the potential cell types from bunch of scRNA-seq data.

Regarding computational future directions, recovering the genes’ heterogeneity over time in individual cells is only a fist step in understanding the complex dynamic processes that drive changes in gene expression. Most scRNA-seq data sets consist of hundreds (and sometimes thousands) of cells that have recently allowed parallel sequencing of substantially larger numbers of cells in an effective manner, which brings additional challenges to the statistical analysis of scRNA-seq data sets (e.g., because of the existence of unknown sub-populations, requiring unsupervised approaches). We expect that developing unified computational methods with time-series single cell gene expression data will yield more biological insights. Inferring the potential types and states of individual cells is thus a useful tool for studying cell differentiation and govern a much wider array of biological processes.

## Methods

### Details of the data sources

We performed our pipeline on both synthetic time series data and real temporal gene expression data downloaded from (GEO). The real time-series scRNA-seq data were obtained from GSE52529 [15]. The data were generated from primary HSMMs, that were cultured in high-serum medium. After a switch to low-serum medium, cells were dissociated and individually captured at 24-h intervals. Ninety-six cells were captured at each of four time points. They original contributors provided a raw FPKM matrix containing 27,429 genes and 372 cells collected at four time points in total. The first step in the single-cell RNA-Seq analysis is identifying poor-quality libraries for further analysis. The gene expression matrix holds the identifiers for genes expressed in at least 80 percent of the total cells in the data set. We then applied the DTWscore to identify specific genes from all pair of cells with time-series expression data. In terms of the simulated strategy, we simulated a set of dynamic gene expression programs to assess the performance of our model on inputs with time-series data. As noted above, time-course experiments fall into four categories, represented by four families of functions. The expression dynamic across ‘pathways’ were drawn from the empirical functions as that in [8]. Genes are classified into two groups. Genes with less variation in the expression levels with time in two cells are labeled with non-heterogeneity as group one. We simulated these gene’s expression values from the same function. The other genes are group two, and the genes with increased biological variability could respond to different kinetics. These genes with heterogeneity labels are derived from different biological processes. Then each gene is entitled with a DTWscore. All the DTWscores are normally distributed. We take the 4 standard deviations above the mean as the threshold for identifying heterogeneous genes. Finally, we call EM algorithm [34] for normal mixture models that define the potential cell types.

$$\begin{aligned} f_{1}(t) & =5c_{1}\cos(t/5)+8+\epsilon_{1} \\ f_{2}(t) & =5c_{2}\sin(t/5)+8+\epsilon_{2} \\ f_{3}(t) & =c_{3}(t/10)^{2}+\epsilon_{3}+5 \\ f_{4}(t) & =5\log(t+1)+8\\ \end{aligned} $$

where *c*_*i*_∼*N*(1,0.01) and *ε*_*i*_∼*N*(0,*σ* ^2^). For the actual values of *t*, we used the sequence range from 0 to 10∗*π* of 314 values. We considered 1000 genes from two types, highly variable genes and non-heterogeneous genes; and two cells from two time series. For each cell, we varied the interval of each different time points, and the corresponding DTWscore is calculated for each gene.

### Details of the DTWscore pipeline

The DTWscore pipeline contains four steps (Fig. 1).

#### Filter low-quality cells

The first step in single-cell RNA-Seq analysis is identifying poor-quality libraries. Most single-cell workflows will include at least some libraries made from dead cells or empty wells in a plate. The expression level of each gene was represented by FPKM values. DTWscore hold the genes expressed in at least 80 percent of the total cells in the data set. Genes that were non-expressed in more than 80 percent of the total cells were excluded, leaving the remaining genes for further analysis. Consequently, thousands of genes could be reduced to hundreds for further analysis.

#### Calculate the average DTWscore for all pair of cells

The DTWscore is calculated based on the FPKM gene expression levels. The dynamic time warping technique [35] is used to detect changes in the expression patterns for time-series scRNA-seq data sets. We assume that two cells are compared, and each cell contains *n*_*p*_(*p*=1,2) temporal gene expression values. Let $X_{ij}^{(p)}$ represents the expression count of gene *i*(*i*=1,…,*N*) of *jth* time points in the *pth* (*p*=1,2) cells. Briefly, if the expression levels of some gene *i* are tracked during the unfolding of a biological process, the process can be conceived as tracing out a trajectory over time. We consider the two temporal gene expression in two cells as two time series: $X_{ij}^{(1)}=(x_{i1}^{(1)},...,x_{im}^{(1)},...)$ and $X_{ij}^{(2)}=(x_{i1}^{(2)},...,x_{in}^{(2)},...)$. We also assume that a non-negative, local dissimilarity function *f* is defined between any pair of elements *x*_*m*_ and *x*_*n*_, with some type of distance:

$$d(m,n)=f(x_{m},x_{n})\geq 0 $$

Note that the most common choice is to assume the Euclidean distance, different definitions (e.g.,those provided by the proxy package [36]) may be useful as well. Thus, the procedure for evaluating the level of differential expression between $X_{ij}^{(1)}$ and $X_{ij}^{(2)}$ involves finding all possible routes through the grid and computing each one’s overall distance, which is defined as the sum of the distances between the individual elements on the warping path [37]. Consequently, the final DTW distance between $X_{ij}^{(1)}$ and $X_{ij}^{(2)}$ is the minimum overall distance over all possible warping paths. The idea underlying DTW is to find the optimal path *ϕ* such that

$$D(X_{ij}^{(1)},X_{ij}^{(2)})=\min\limits_{\phi} d_{\phi}(X_{ij}^{(1)},X_{ij}^{(2)}) $$

The DTW algorithm makes use of dynamic programming and works by keeping track of the cost of the best path at each point in the grid:

$$\begin{aligned} \gamma(1,1) &=d(1,1)\\ \gamma(m,1) & =d(m,1)+\gamma(m-1,1)\\ \gamma(1,n) & =d(1,n)+\gamma(1,n-1)\\ \gamma(m,n) & =d(m,n)+\min(\gamma(m,n-1),\\&\gamma(m-1,n-1),\gamma(m-1,n)) \end{aligned} $$

Consequently, $D(X_{ij}^{(1)},X_{ij}^{(2)})=\gamma (n_{1},n_{2})/(n_{1}+n_{2})$. During the calculation process of the DTW grid, it is not actually known which path minimizes the overall distance, but this path can be traced back when the end point is reached. We observed that the DTW distances are strongly correlated with maximum gene expression levels; therefore, a normalizing procedure was necessary. We used the R package named dtw [38], which provides both distance and normalized distance for further analysis.

#### Identify highly variable genes with a model-based threshold

Model-based threshold to identify highly variable genes will change significantly among various types of datasets. As the variabilities are high in scRNA-seq time-series data, a fixed threshold for the DTWscore is less effective in many settings. Flexible thresholds for the DTWscore are necessary, allowing the test of variabilities in response to a numerically estimated trend in the predictors, alleviating the burden of specifying their distribution. We utilized the distribution model to identify the specific gene sets for further analysis. We briefly summarize the main insight. As noted above, the empirical distribution of the DTWscore from all time-course datasets falls into normal distribution (Fig. 7). Probability density function could be achieved by the estimation of mean and standard deviation values. Probability density that equals to 0.95 is defined as the default threshold for selecting genes and genes with DTWscores larger than the threshold are then classified as differentially expressed between two single cells. Those genes could be served as potential biomarkers that track some disease process by the researchers. Following the previous section, the DTWscore *D*_*i*_,(*i*=1,…,*N*)for each gene is obtained. Suppose

$$D_{i} \sim N(\mu, \sigma^{2}) $$

where

$$\mu = \sum_{i=1}^{N} D_{i}, \sigma^{2} = \sum_{i=1}^{N} (D_{i} - \mu)^{2}/N $$

#### Classify cells of different types by normal mixture model

We can classify cells as follows. First, to cluster the cells, we choose the gene with the highest DTWscore and make full use of its expression values of all the time points. Next procedure requires R package mclust which provides Gaussian finite mixture model fitted by EM algorithm [34]. The roc plot indicates the result of our classification is good. Overall, we have successfully classified all the cells. As noted above, the empirical distribution of the DTWscore from all time-course datasets falls into two normal distribution (Fig. 8). Each type of experiment has a characteristic expression outcome. However, these distinct outcomes are achieved by two types of empirical distribution. the 4 standard deviations above the mean is defined as the default threshold for selecting genes and genes with DTWscores larger than the threshold are then classified as differentially expressed between two single cells. Those genes could then subjected to cell cluster analysis by the researchers. Combinations of these basic outcomes result in the flexible application of the DTWscore methods. In future work, we will explore more conditions from various datasets.

## Additional files

Additional file 1 **Figure S1.** DTWscore identifies heterogeneous genes and non-heterogeneous genes from the synthetic data (condition 3). (PDF 122 kb)

Additional file 2 **Figure S2.** DTWscore identifies heterogeneous genes and non-heterogeneous genes from the synthetic data (condition 4). (PDF 122 kb)

Additional file 3 **Figure S3.** DTWscore identifies heterogeneous genes and non-heterogeneous genes from the synthetic data (condition 5). (PDF 122 kb)

Additional file 4 **Figure S4.** DTWscore identifies heterogeneous genes and non-heterogeneous genes from the synthetic data (condition 6). (PDF 122 kb)

Additional file 5 **Figure S5.** Model-based clustering of HSMM dataset by any two highly variable genes. (PDF 40 kb)

Additional file 6 **Figure S6.** Model-based clustering of HSMM dataset by any three highly variable genes. (PDF 68 kb)

## Abbreviations

DTW: Dynamic Time Wraping
scRNA-seq: Single Cell RNA Sequencing
DE genes: Differential Expressed Genes
