# Supplementary figures and images for: DTWscore: differential expression and cell clustering analysis for time-series single-cell RNA-seq data
Source: BMC Bioinformatics. 2017 May 23;18:270. doi: 10.1186/s12859-017-1647-3 (PMC5442705; doi:10.1186/s12859-017-1647-3)

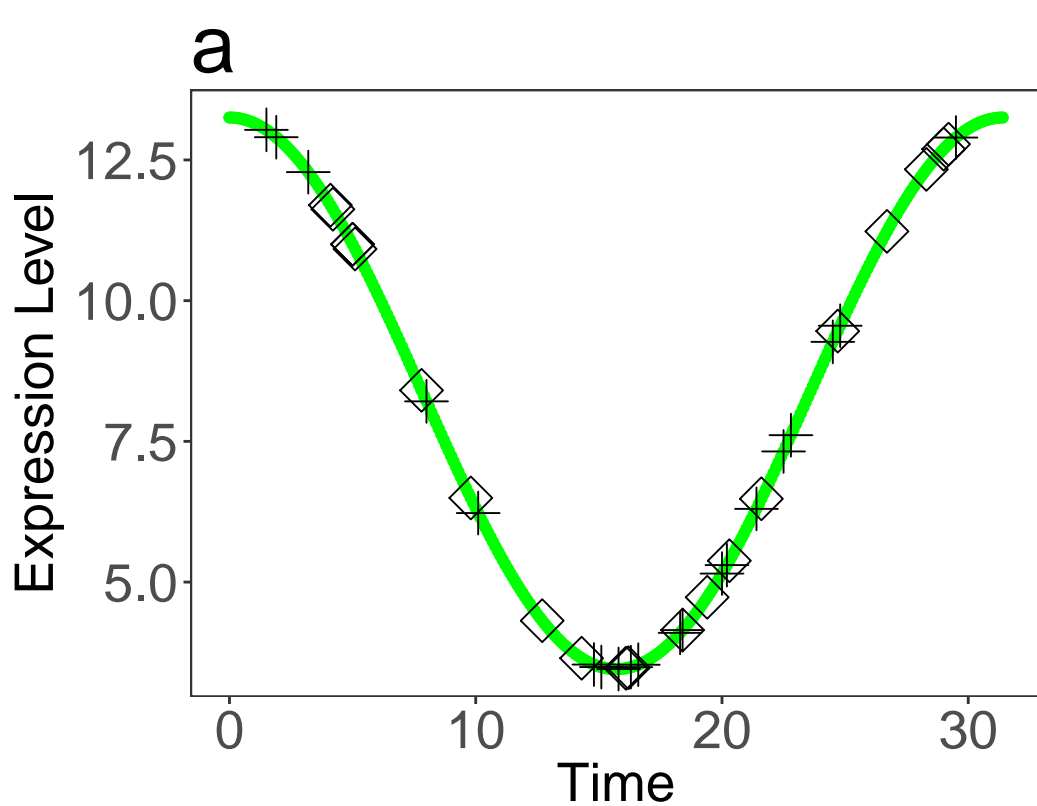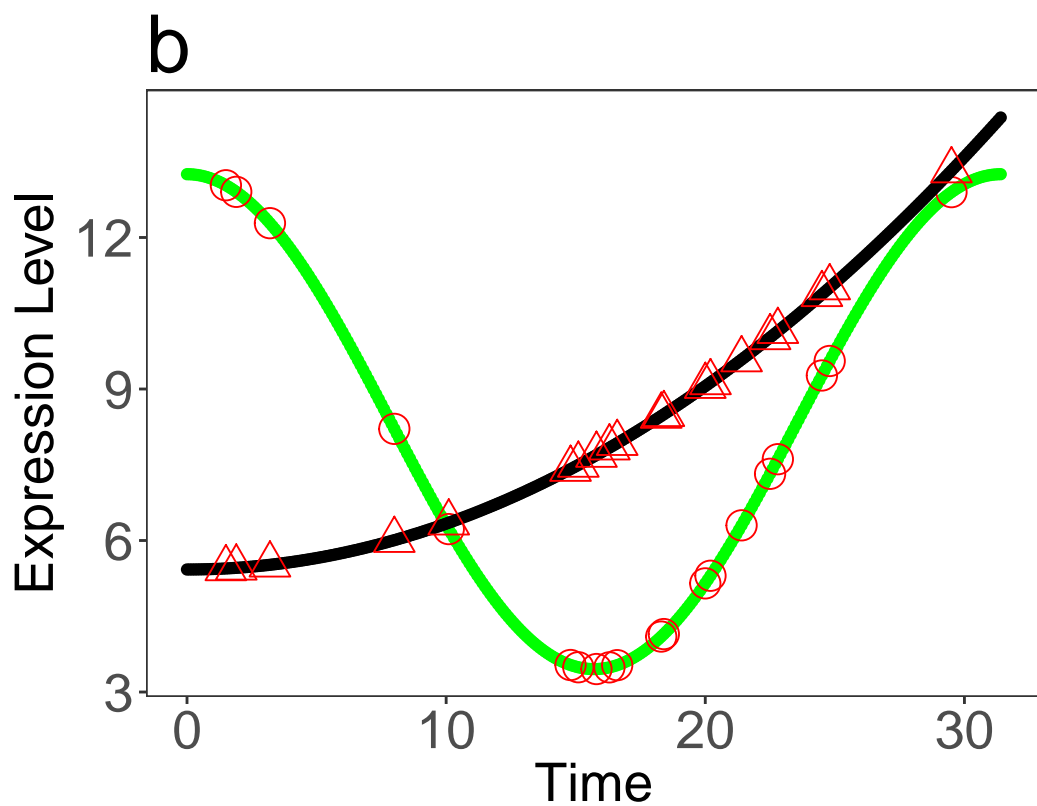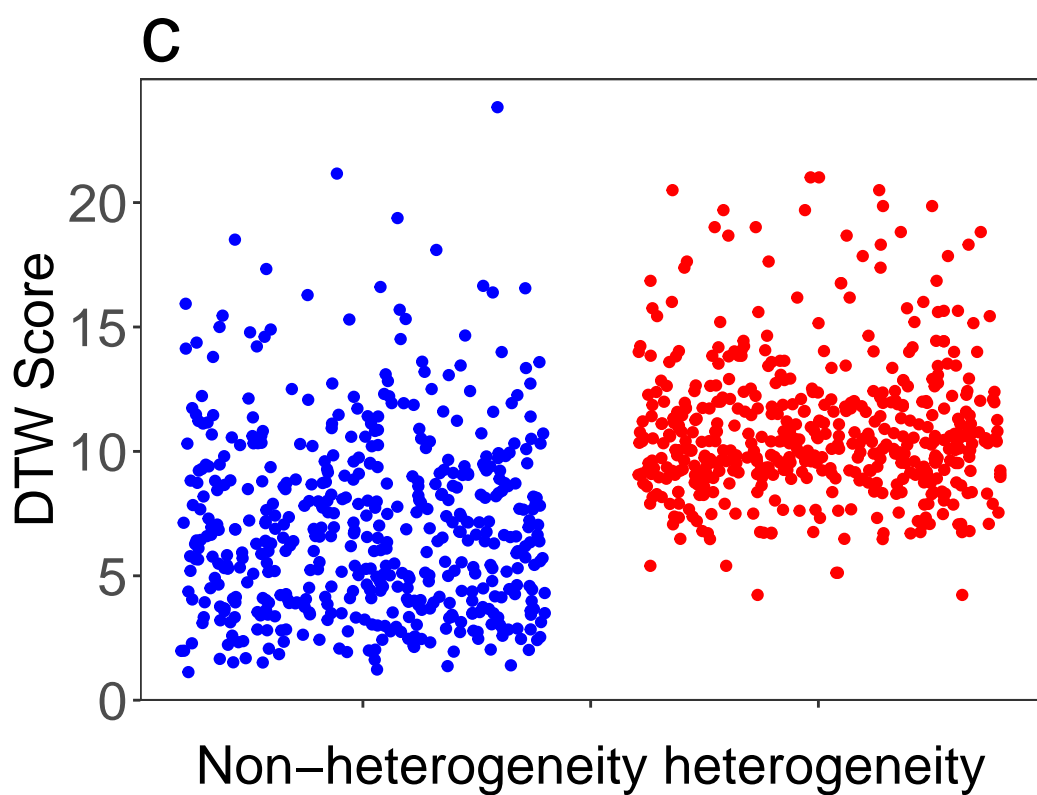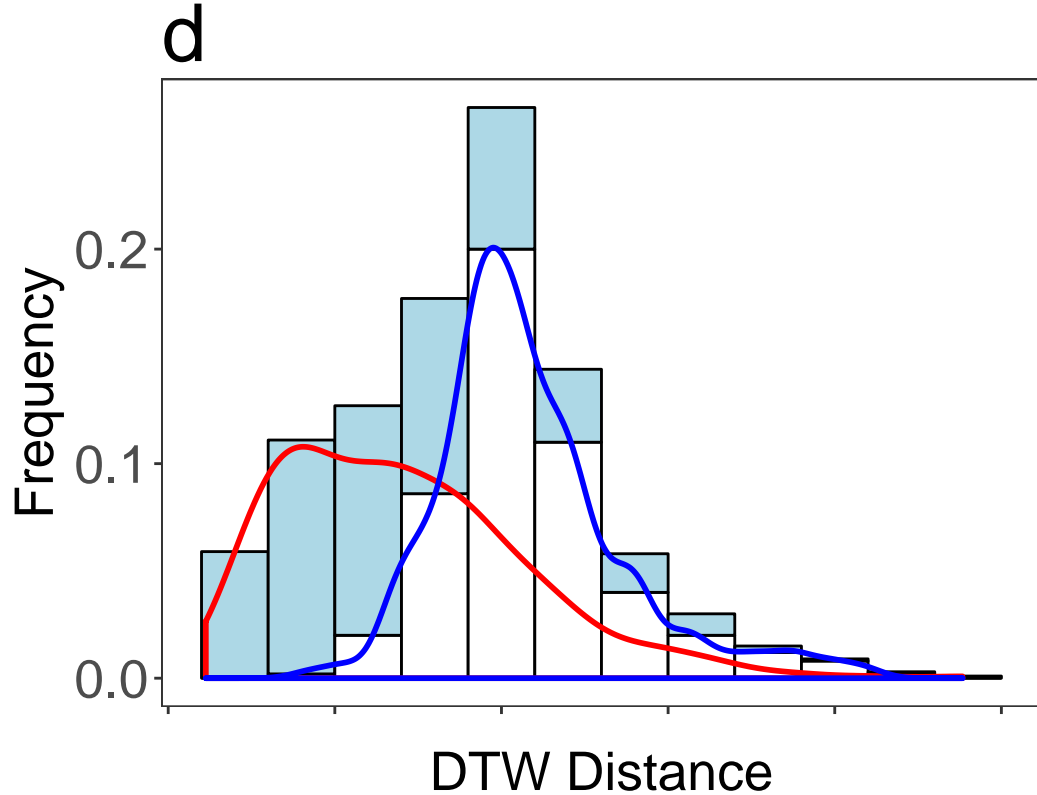

Supplement: Supplementary file 1 — Figure S1. DTWscore identifies heterogeneous genes and non-heterogeneous genes from the synthetic data (condition 3). (PDF 122 kb) [file 12859_2017_1647_MOESM1_ESM.pdf]

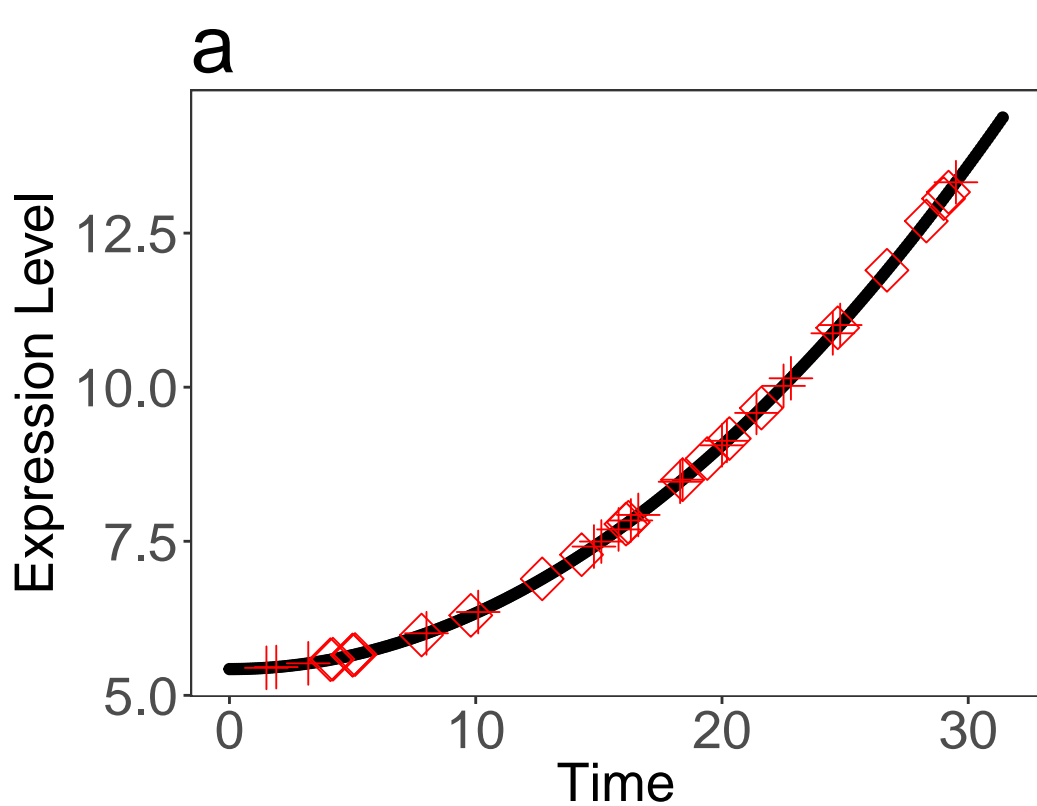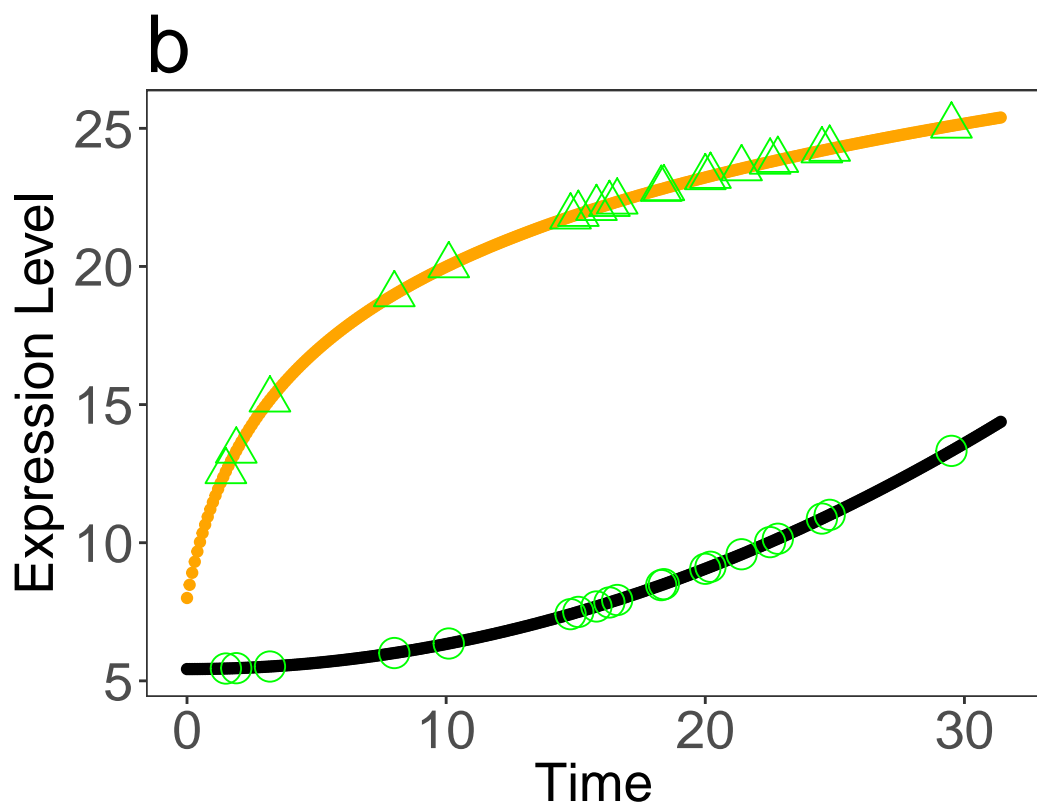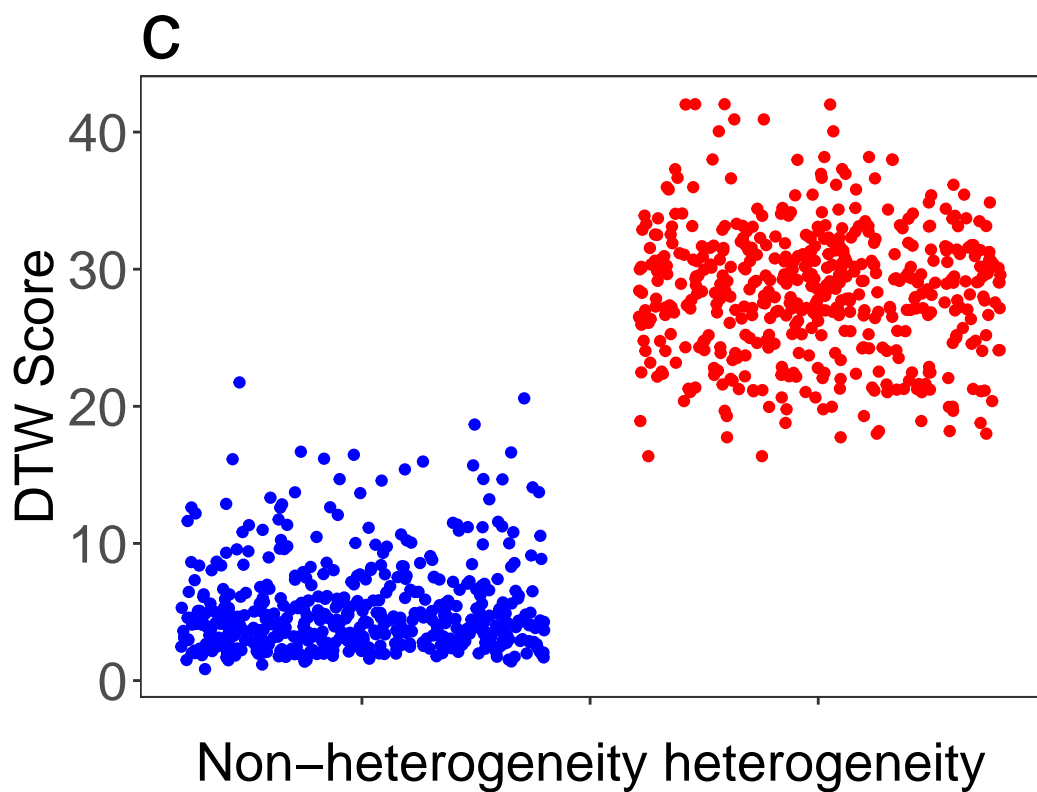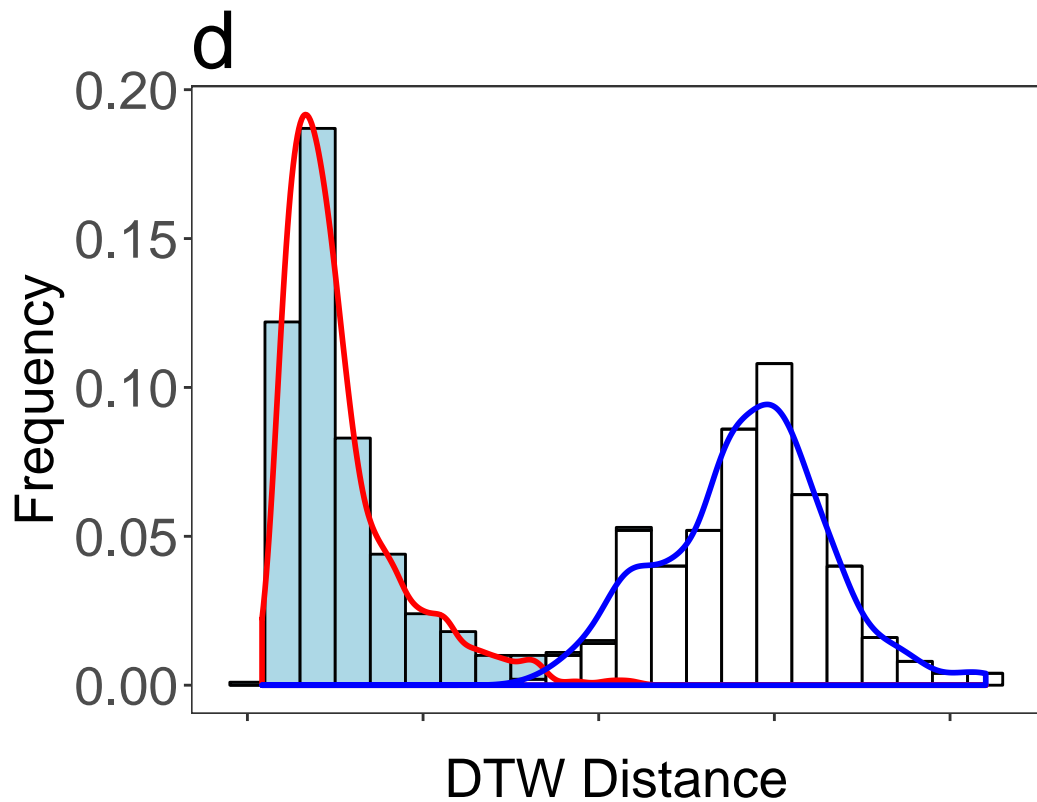

Supplement: Supplementary file 2 — Figure S2. DTWscore identifies heterogeneous genes and non-heterogeneous genes from the synthetic data (condition 4). (PDF 122 kb) [file 12859_2017_1647_MOESM2_ESM.pdf]

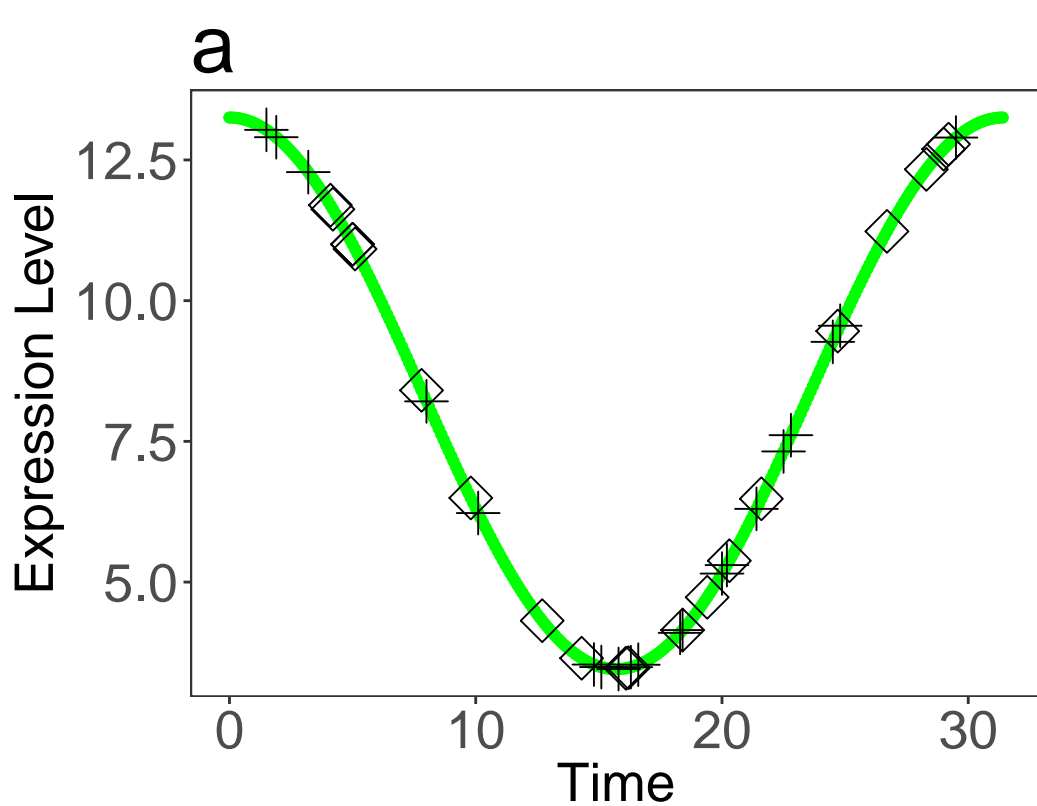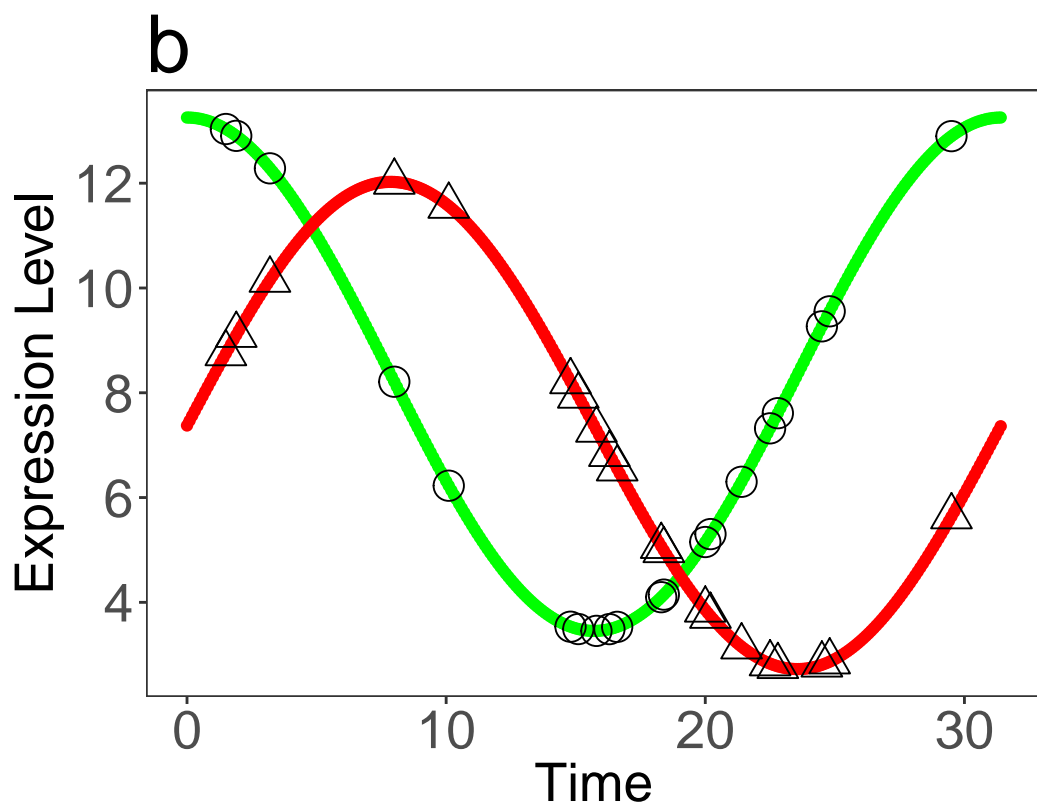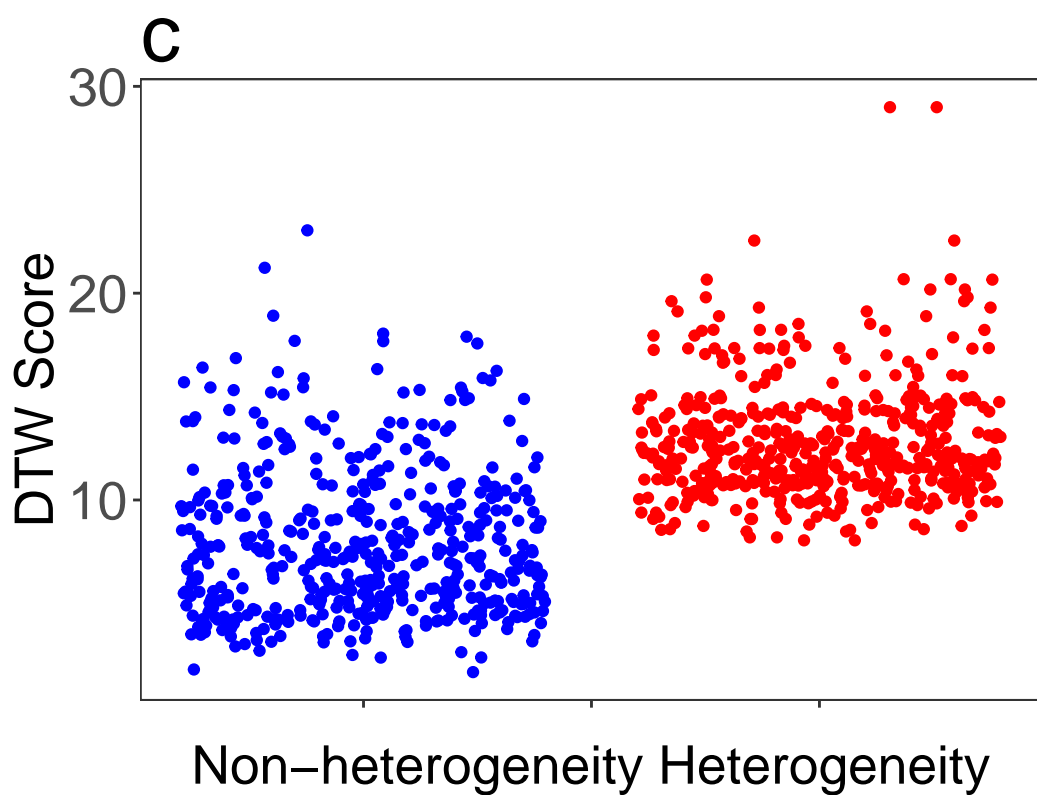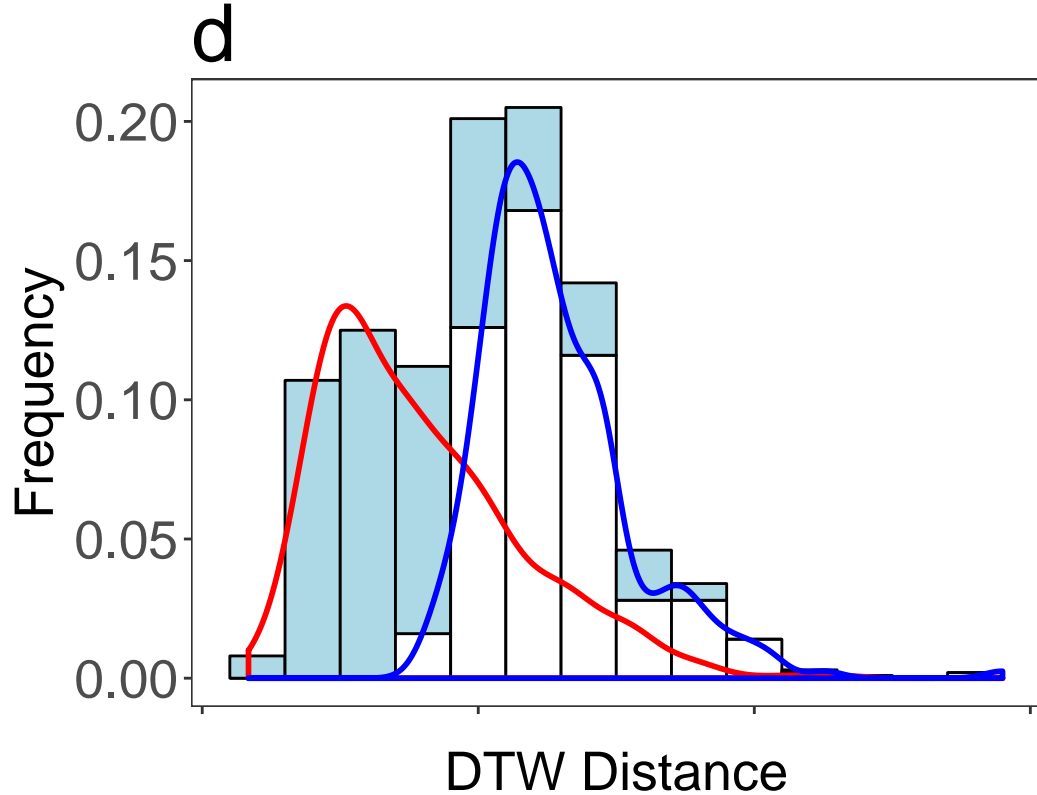

Supplement: Supplementary file 3 — Figure S3. DTWscore identifies heterogeneous genes and non-heterogeneous genes from the synthetic data (condition 5). (PDF 122 kb) [file 12859_2017_1647_MOESM3_ESM.pdf]

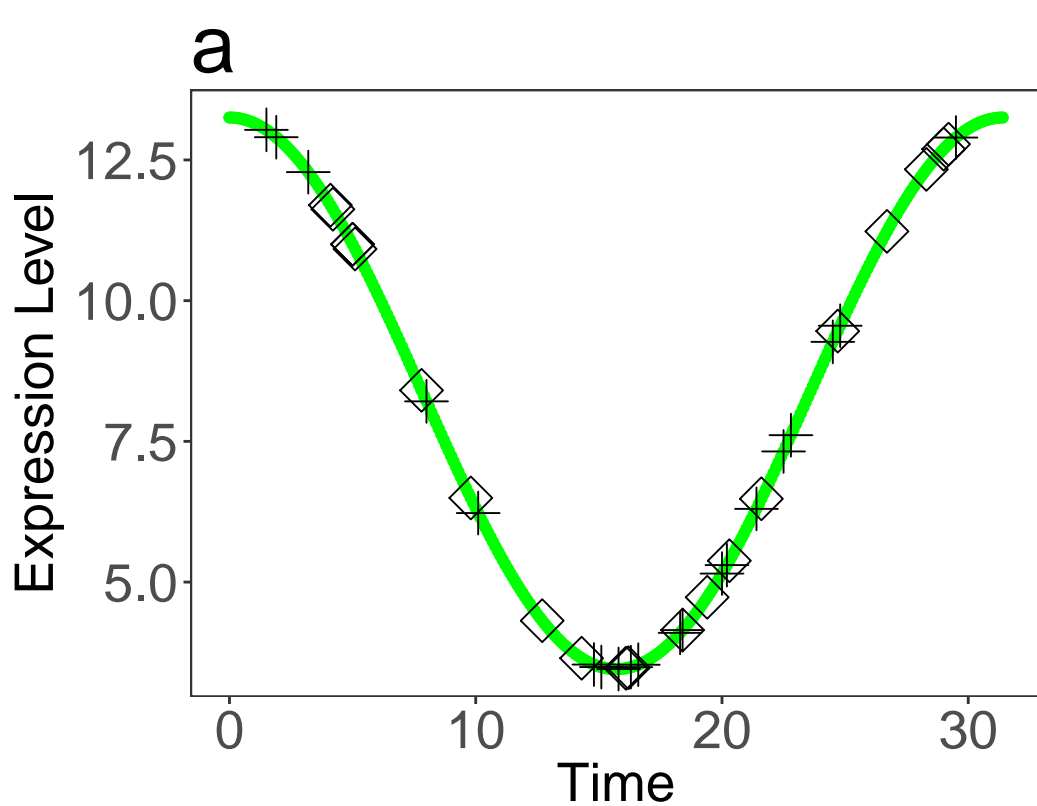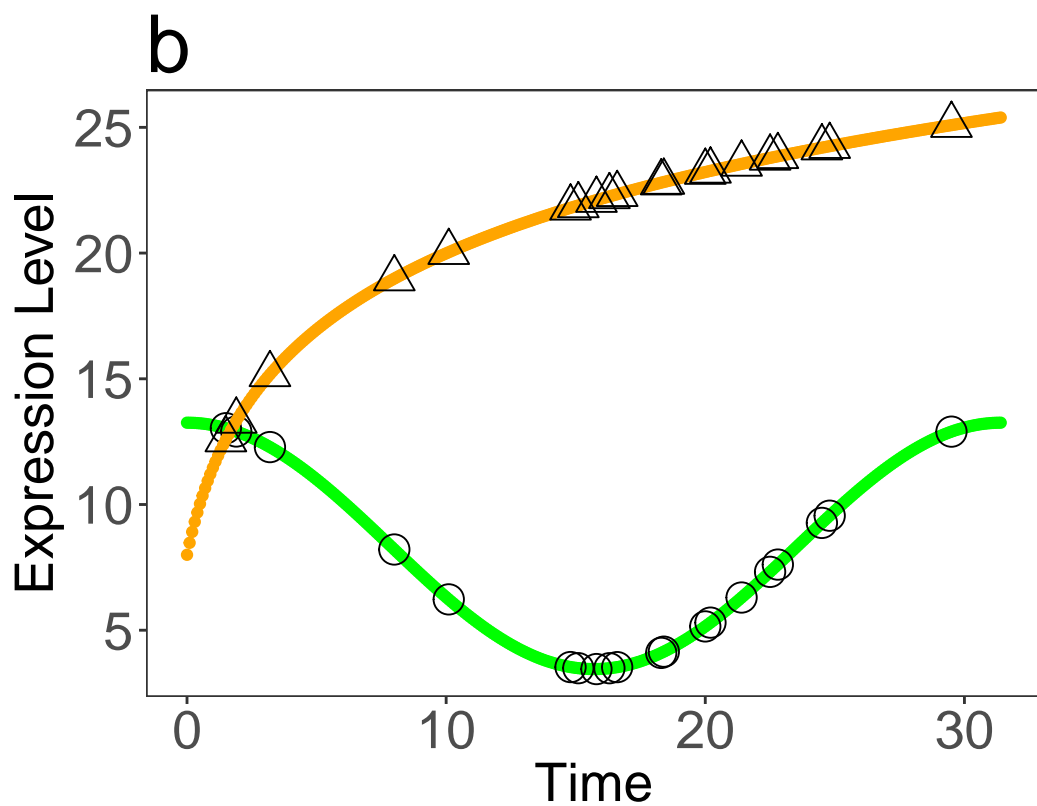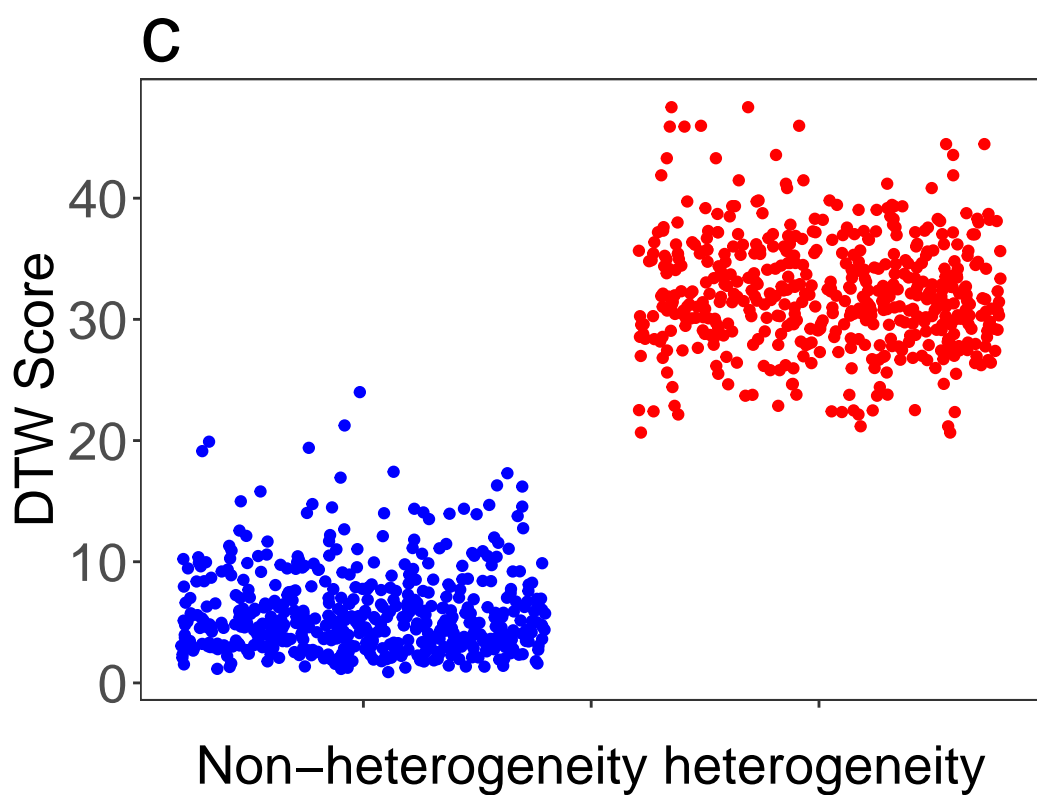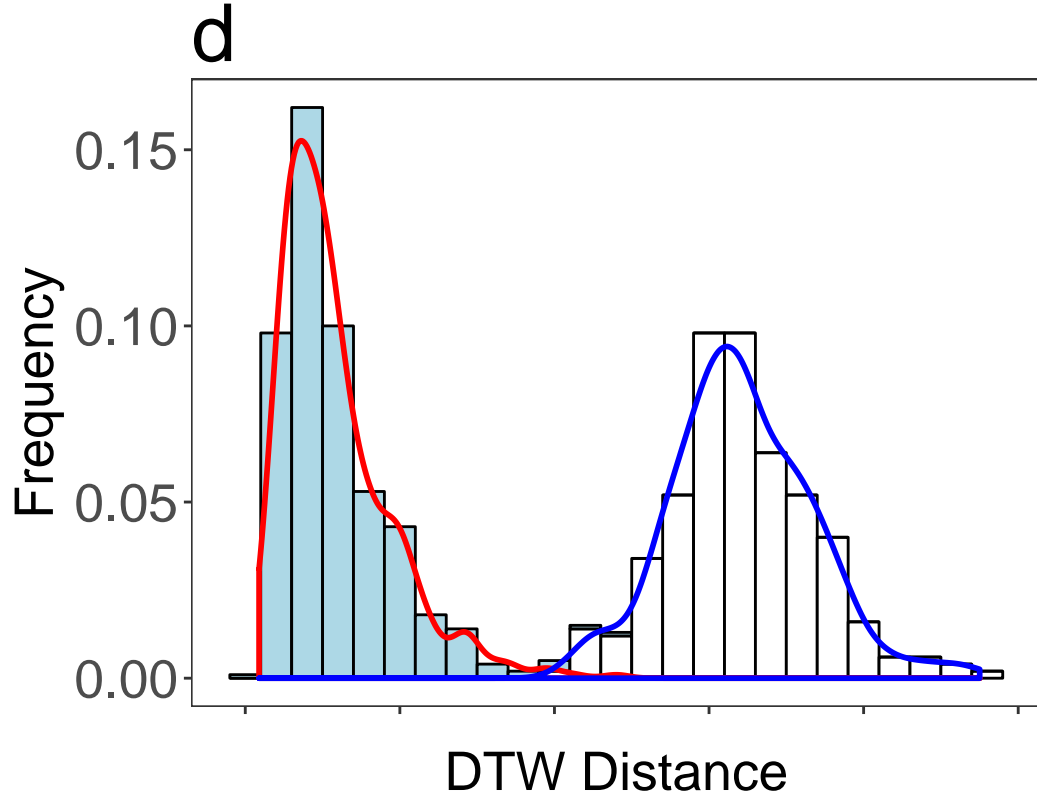

Supplement: Supplementary file 4 — Figure S4. DTWscore identifies heterogeneous genes and non-heterogeneous genes from the synthetic data (condition 6). (PDF 122 kb) [file 12859_2017_1647_MOESM4_ESM.pdf]

**Classification**

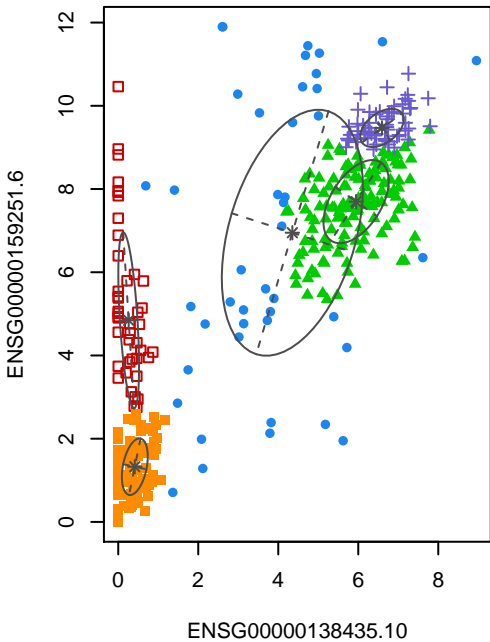

**Classification**

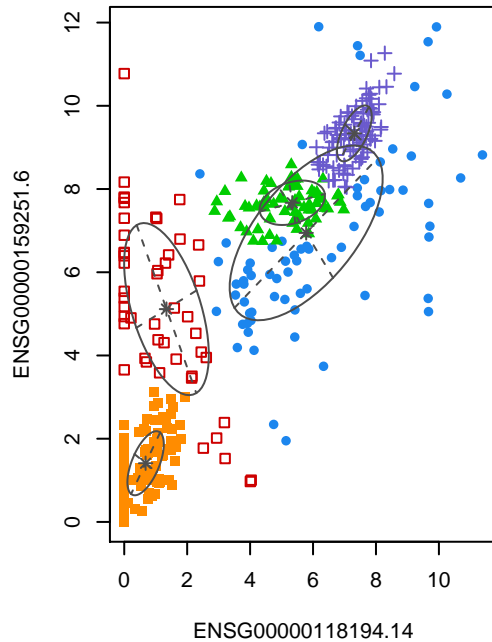

**Classification**

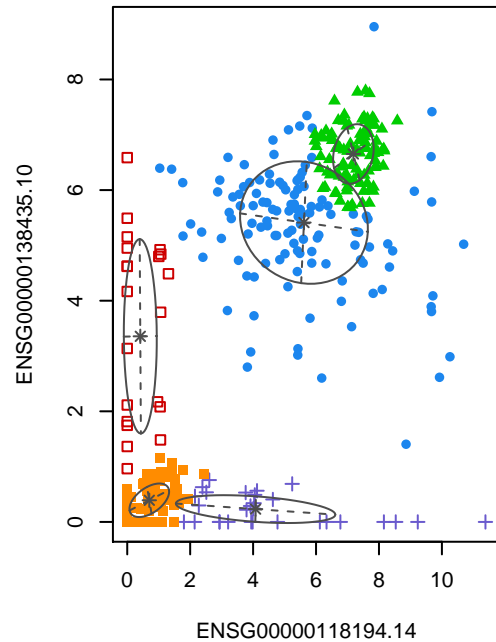

Supplement: Supplementary file 5 — Figure S5. Model-based clustering of HSMM dataset by any two highly variable genes. (PDF 40 kb) [file 12859_2017_1647_MOESM5_ESM.pdf]

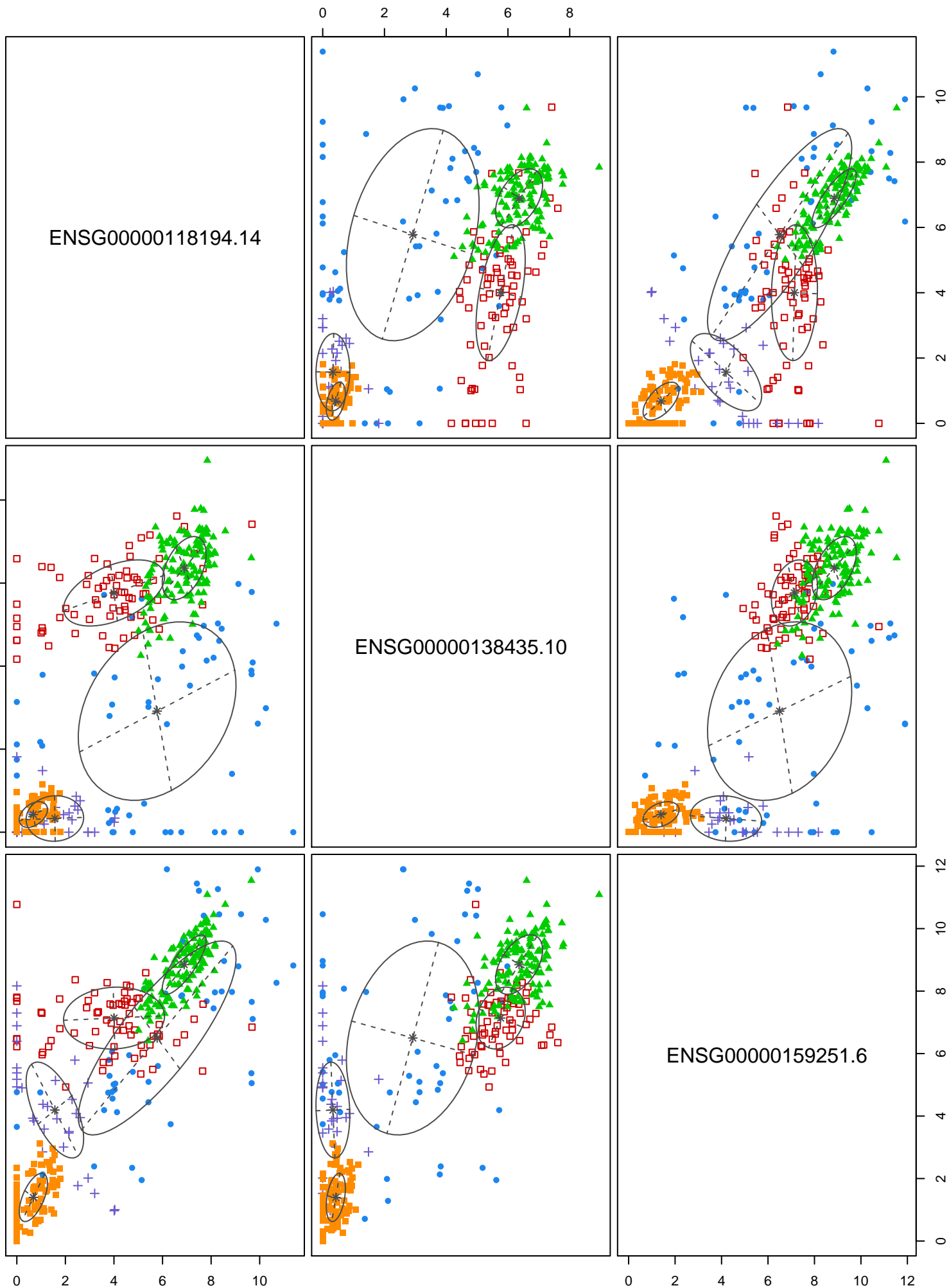

Supplement: Supplementary file 6 — Figure S6. Model-based clustering of HSMM dataset by any three highly variable genes. (PDF 68 kb) [file 12859_2017_1647_MOESM6_ESM.pdf]
